# Supplementary material for: Solar overall water-splitting by a spin-hybrid all-organic semiconductor
Source: Nat Commun. 2024 Jun 13;15:5047. doi: 10.1038/s41467-024-49511-7 (PMC11176302; doi:10.1038/s41467-024-49511-7)
Supplement: Supplementary file 1 — Supplementary Information [file 41467_2024_49511_MOESM1_ESM.pdf]

Supplementary

Solar overall water-splitting by a spin-hybrid all-organic semiconductor

Xinyu Lin[1], Yue Hao[1], Yanjun Gong[2], Peng Zhou[3], Dongge Ma[4], Zhonghuan Liu[1], Yuming Sun[1], Hongyang Sun[1], Yahui Chen[1], Shuhan Jia[1], Wanhe Li[1], Chengqi Guo[1], Yiying Zhou[1], Pengwei Huo[1], Yan Yan[1*], Wanhong Ma[2*], Shouqi Yuan[1*], Jincai Zhao[2]

[1]*School of Chemistry & Chemical Engineering/Research Center of Fluid Machinery Engineering and Technology, Jiangsu University, Zhenjiang, China, 212013.*

[2]*Key Laboratory of Photochemistry, Institute of chemistry, Chinese Academy of Sciences, Beijing, China, 100190; University of Chinese Academy of Sciences, Beijing, China, 100049.*

[3]*Electrical Engineering & Computer Science, University of Michigan, Michigan, USA, 48109-2122.*

[4]*Department of Chemistry, College of Chemistry and Materials Engineering, Beijing Technology and Business University, Beijing, China, 100048.*

Corresponding authors:

Prof. Yan Yan (dgy5212004@163.com)

Prof. Wanhong Ma (whma@iccas.ac.cn)

Prof. Shouqi Yuan (shouqiy@ujs.edu.cn)

Supplementary Figures

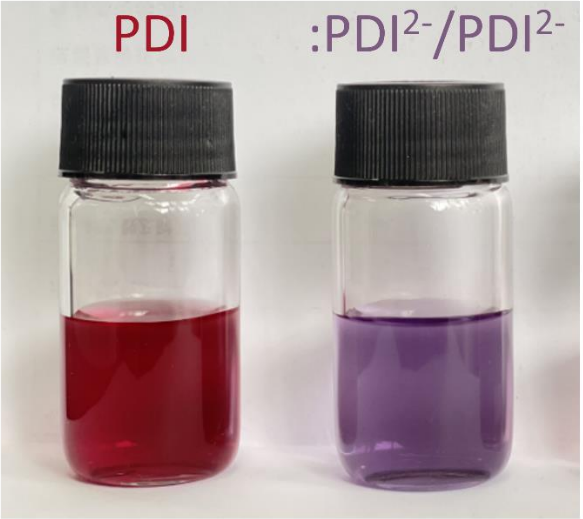

Supplementary Fig. 1. Sample comparison. Photographs of 0.04 g L^{-1} PDI and $:PDI^{2-}/PDI^{2-}$ aqueous solution.

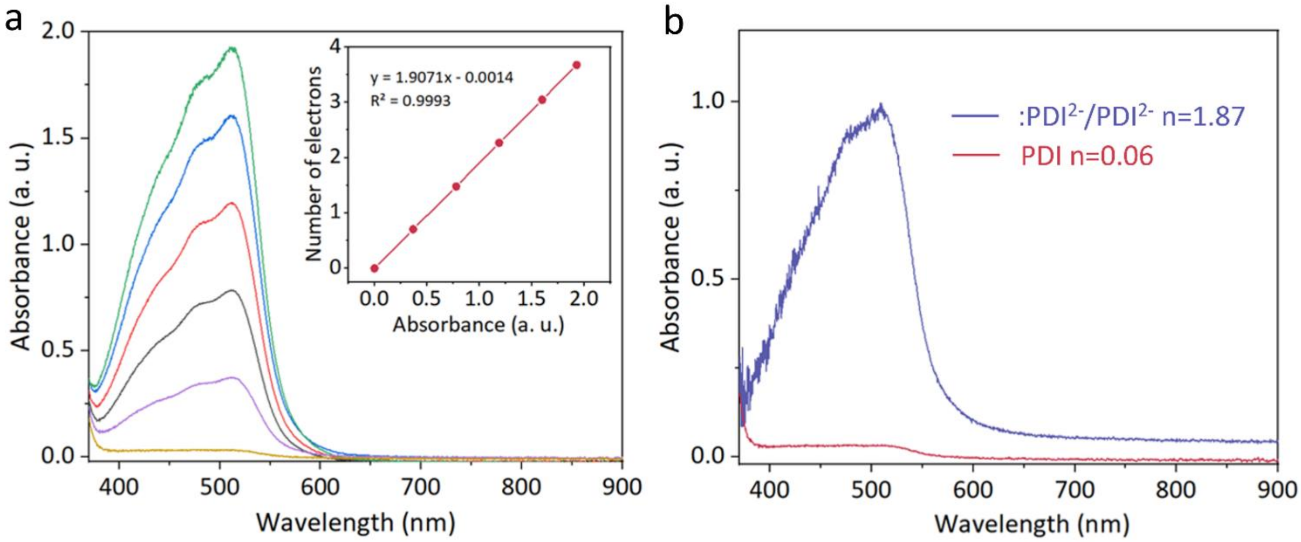

Supplementary Fig. 2. Fe(III)-1, 10-Phenanthroline spectrometric titration measurements. (a) Standard curves for Fe(III)-1, 10-phenanthroline back titration measurements. **(b)** UV-vis absorption spectra of Fe(II)-1, 10-phenanthroline after the titration of reductive electrons on PDI and $:PDI^{2-}/PDI^{2-}$.

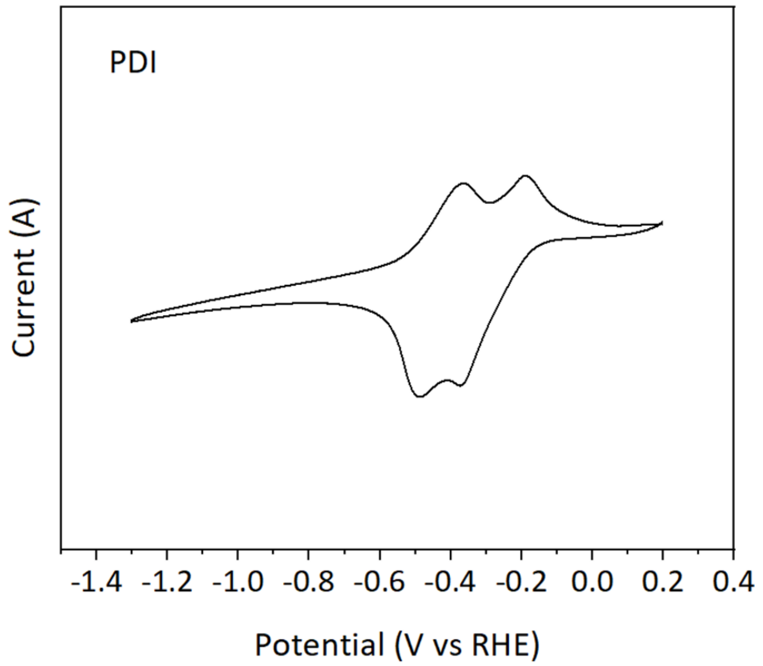

Supplementary Fig. 3. Cyclic voltammetry curves of PDI. The polarization curves in 0.15 M TBATFB/DMSO at a scan rate is 10 mV s^{-1}, and the loading amounts of all catalysts on carbon papers are 0.5 mg cm^{-2}.

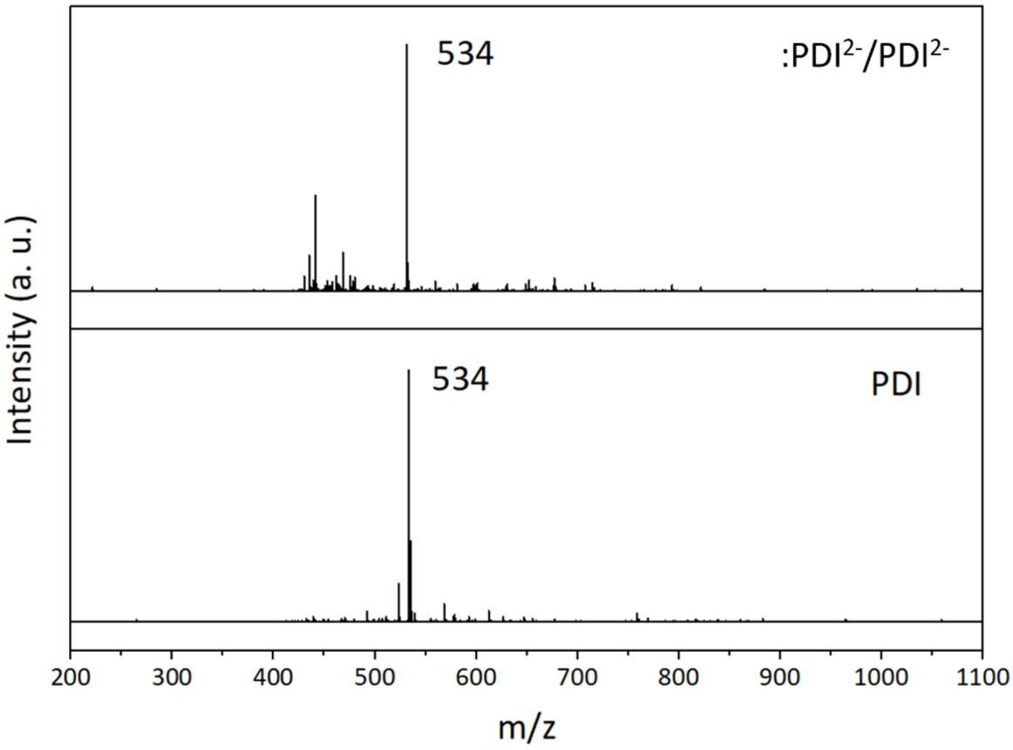

Supplementary Fig. 4. Comparison of molecular weight of samples. Mass spectra of PDI and :PDI^{2-}/PDI^{2-}.

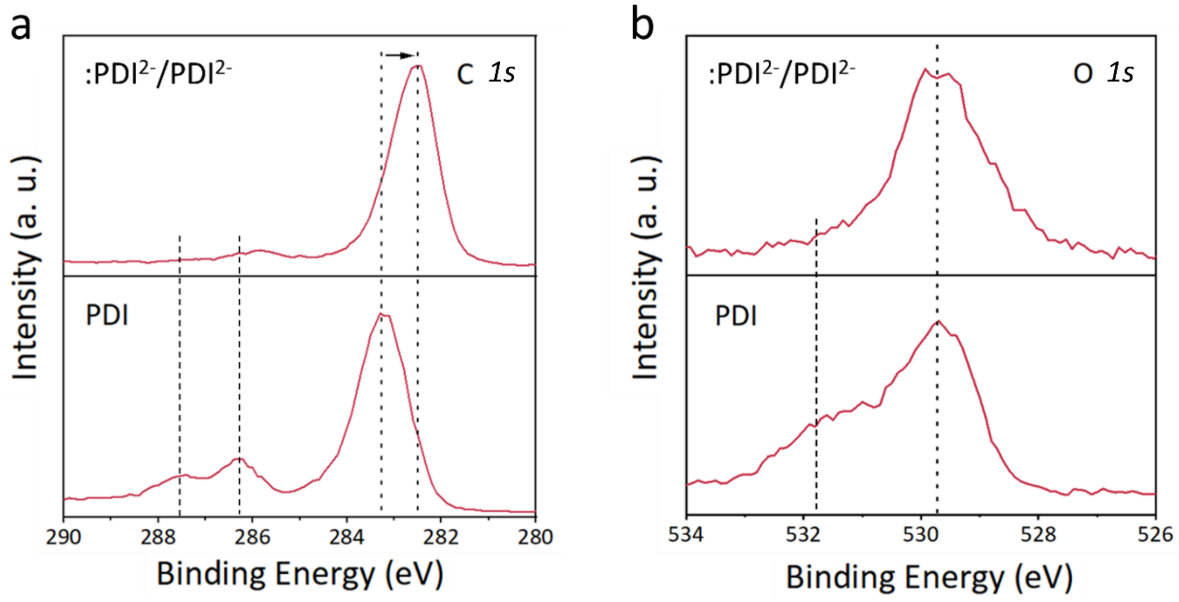

Supplementary Fig. 5. NAP-XPS. (a) C 1s and **(b)** O1s spectra of PDI and :PDI^{2-}/PDI^{2-}.

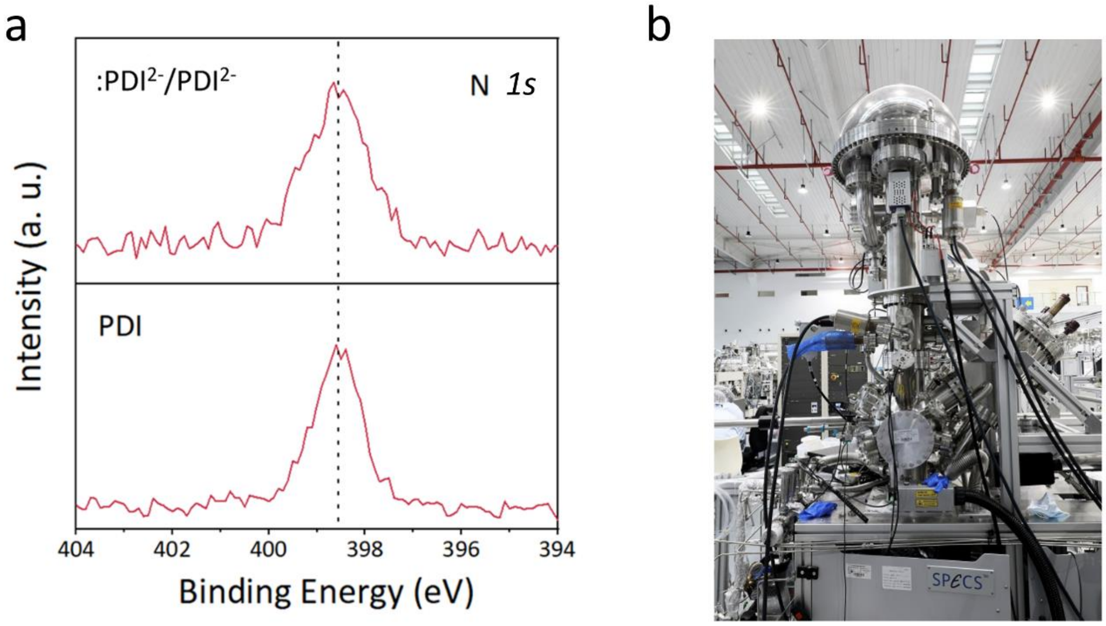

Supplementary Fig. 6. NAP-XPS. (a) N 1s spectra of PDI and :PDI^{2-}/PDI^{2-} and **(b)** image of equipment.

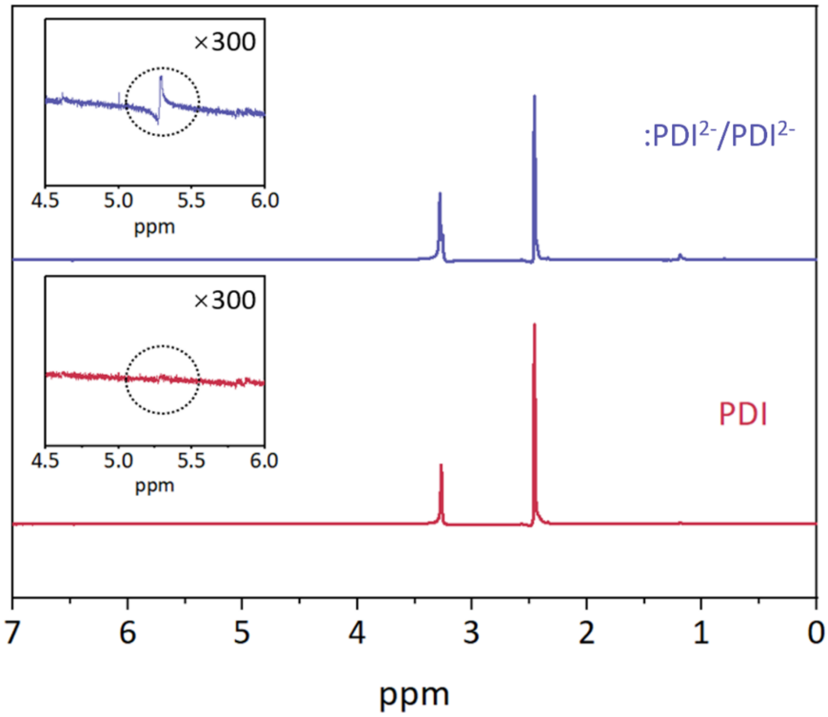

Supplementary Fig. 7. Comparison of sample structures. ^{1}H NMR spectra of PDI and $:PDI^{2-}/PDI^{2-}$.

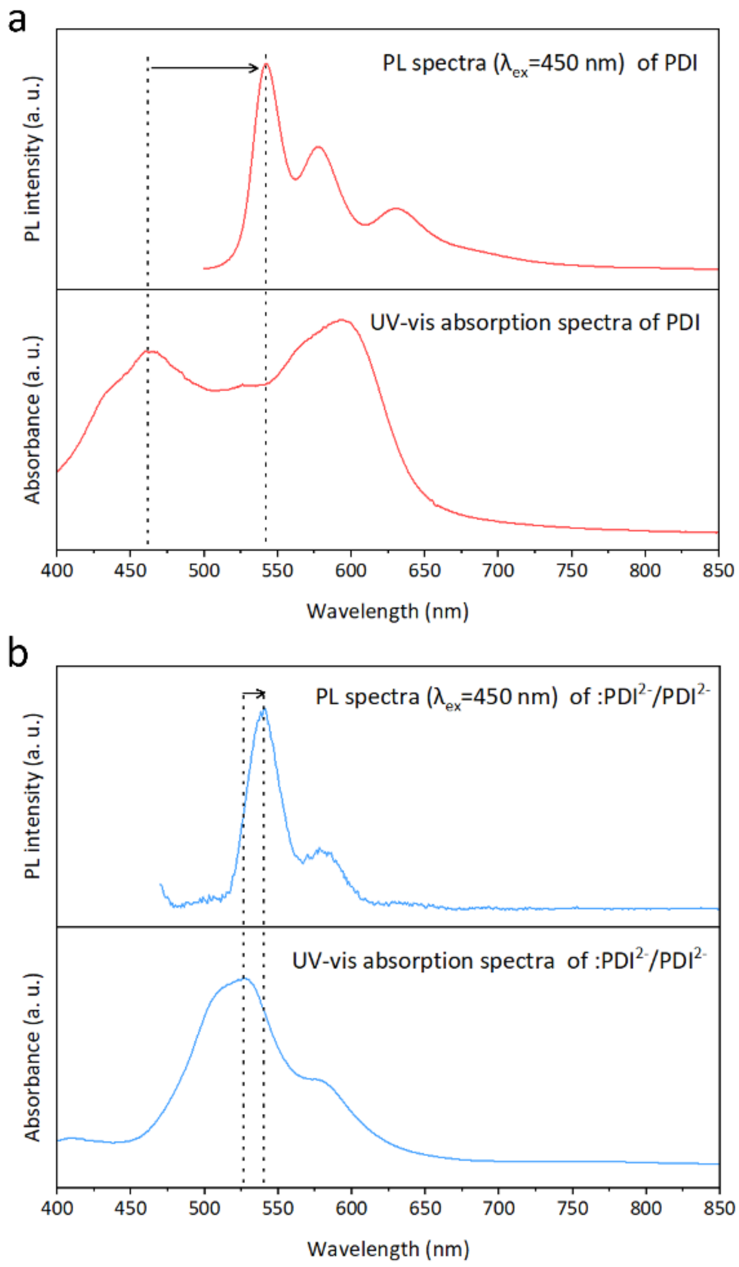

Supplementary Fig. 8. Comparison of stokes shift of samples. UV-vis absorption and PL emission spectra of **(a)** PDI and **(b)** :PDI^{2-}/PDI^{2-}. The Stokes shift is marked.

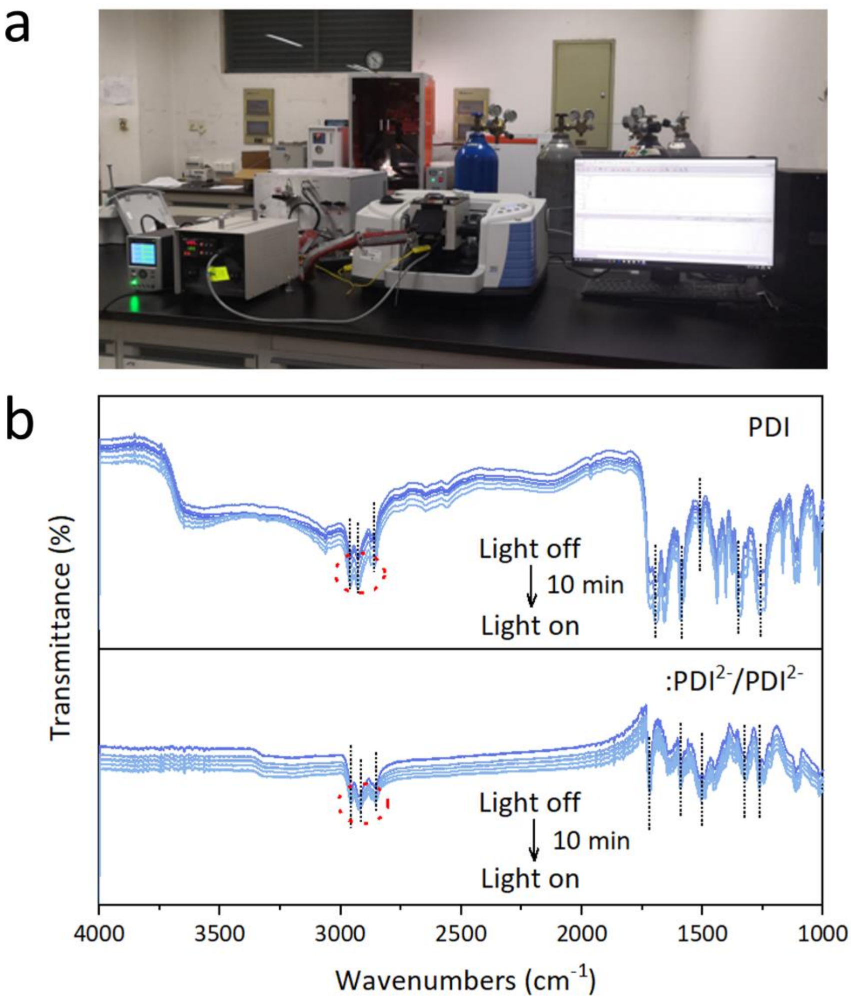

Supplementary Fig. 9. Infrared absorption spectra on different samples. (a) Image of in-situ FT-IR equipment and **(b)** In-situ FT-IR spectra of PDI and :PDI^{2-}/PDI^{2-} under constant white-light (300W Xe-lamp) irradiation.

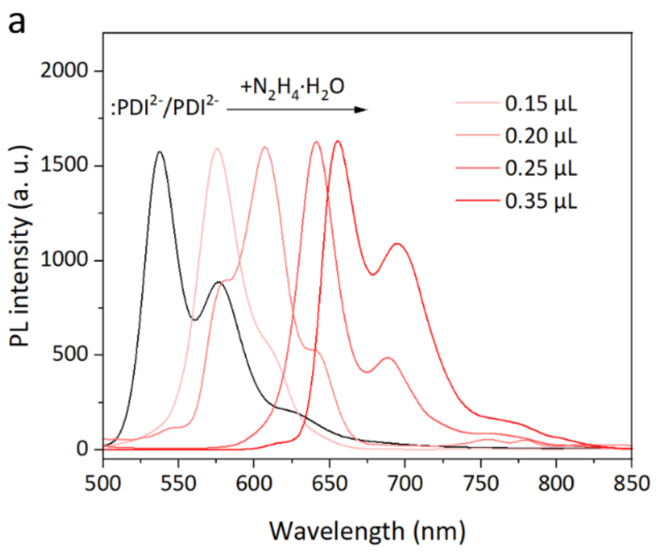

Supplementary Fig. 10. Control experiments show PL spectra under different conditions. (a) PL spectra (λ_{ex}=450 nm) of isolated :PDI^{2-}/PDI^{2-} DMF solution in addition of different amount of hydrazine hydrate. **(b)** PL spectrum (λ_{ex}=450 nm) spectra (λ_{ex}=450 nm) of isolated :PDI^{2-}/PDI^{2-} DMF solution in addition of 0.35 µL ammonia, DEA, and TEOA.

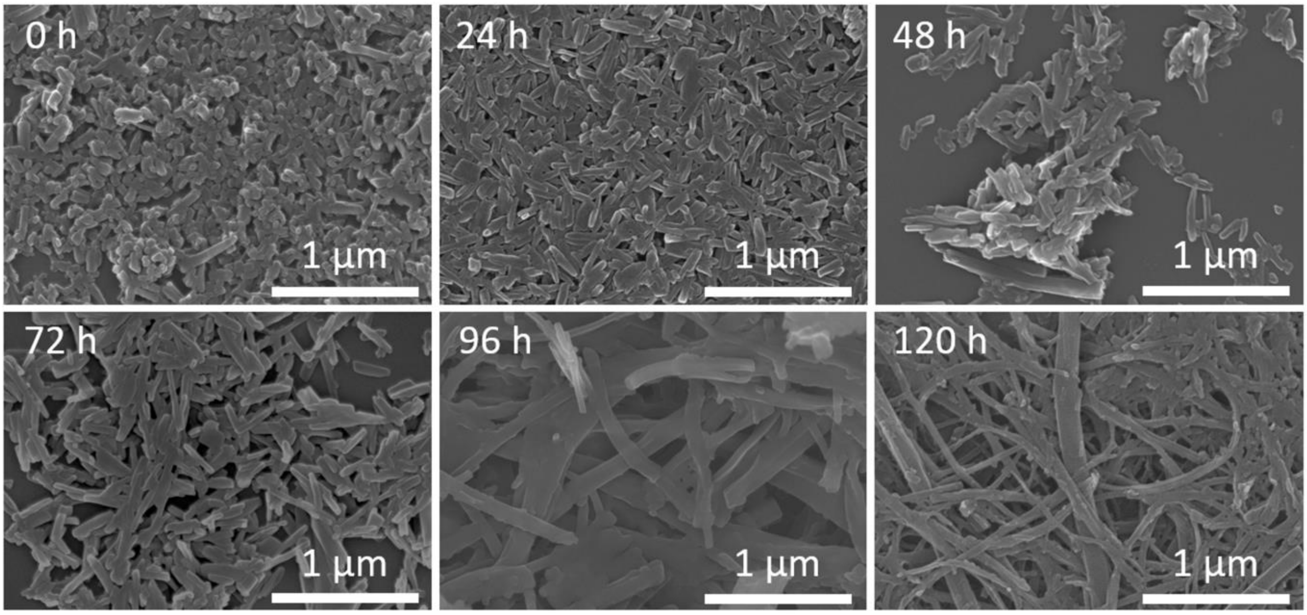

Supplementary Fig. 11. Morphological changes of PDI during self-assembly process. SEM images to show the morphology evolution of pristine PDI during the self-assembly.

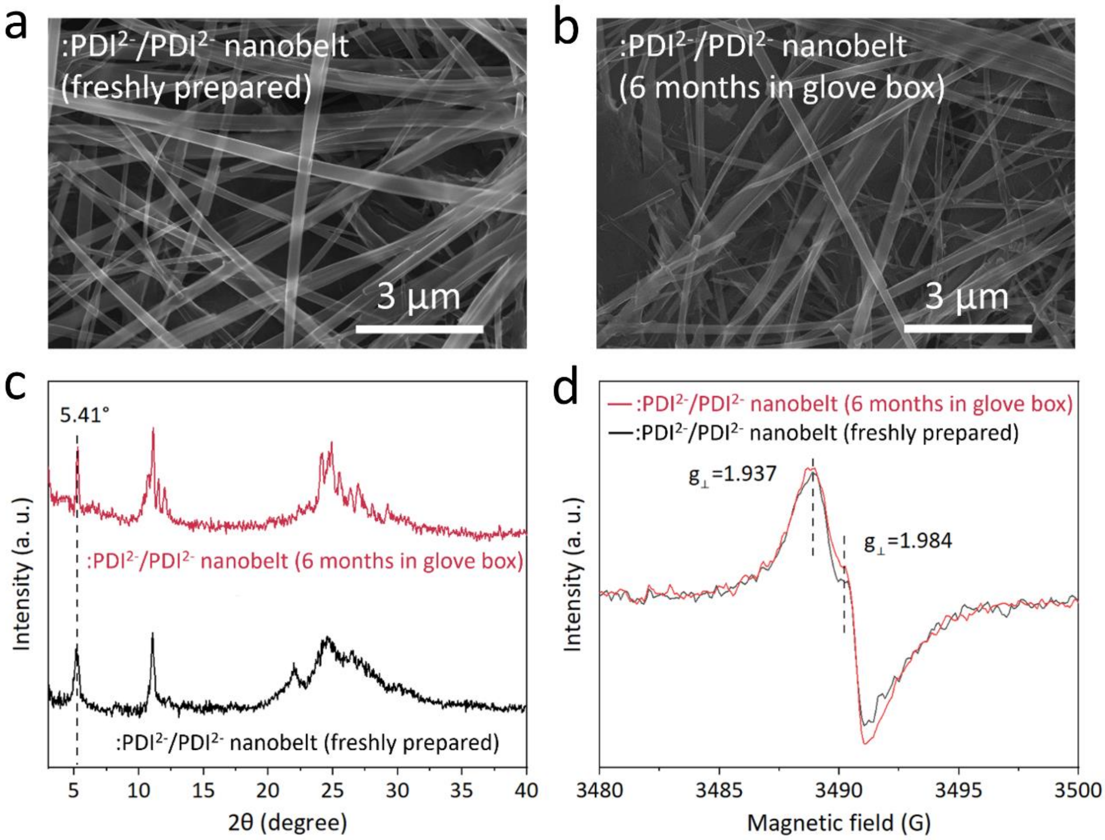

Supplementary Fig. 12. Stability of the sample. SEM images of **(a)** freshly prepared :PDI^{2-}/PDI^{2-} nanobelt (120 h) and **(b)** :PDI^{2-}/PDI^{2-} nanobelt stored in N_2 glove box for 6 months. **(c)** XRD patterns of :PDI^{2-}/PDI^{2-} nanobelt (120 h) freshly prepared and stored in N_2 glove box for 6 months. **(d)** ESR spectra of :PDI^{2-}/PDI^{2-} nanobelt (120 h) freshly prepared and stored in N_2 glove box for 6 months.

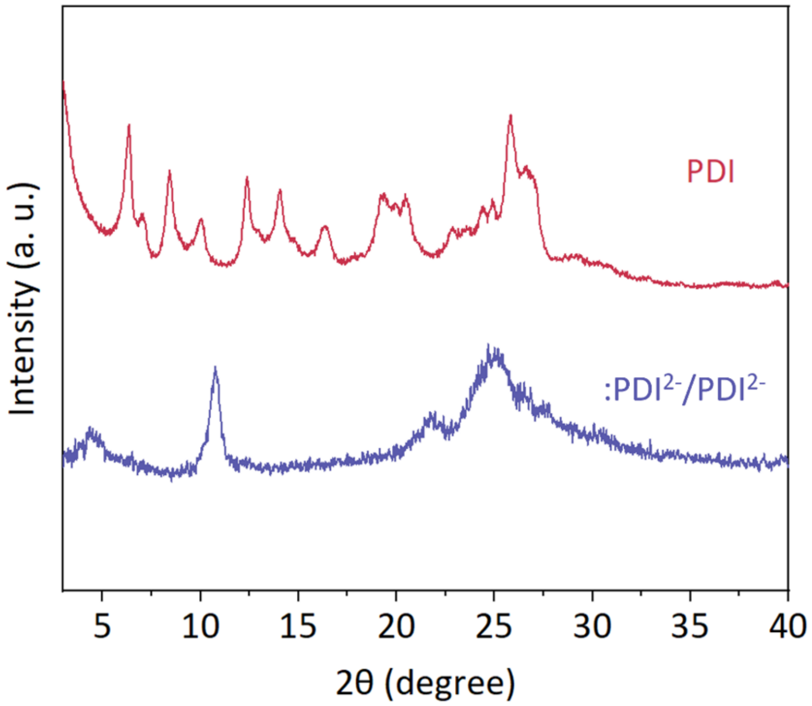

Supplementary Fig. 13. Comparison of crystal structures. XRD patterns of PDI and :PDI^{2-}/PDI^{2-}.

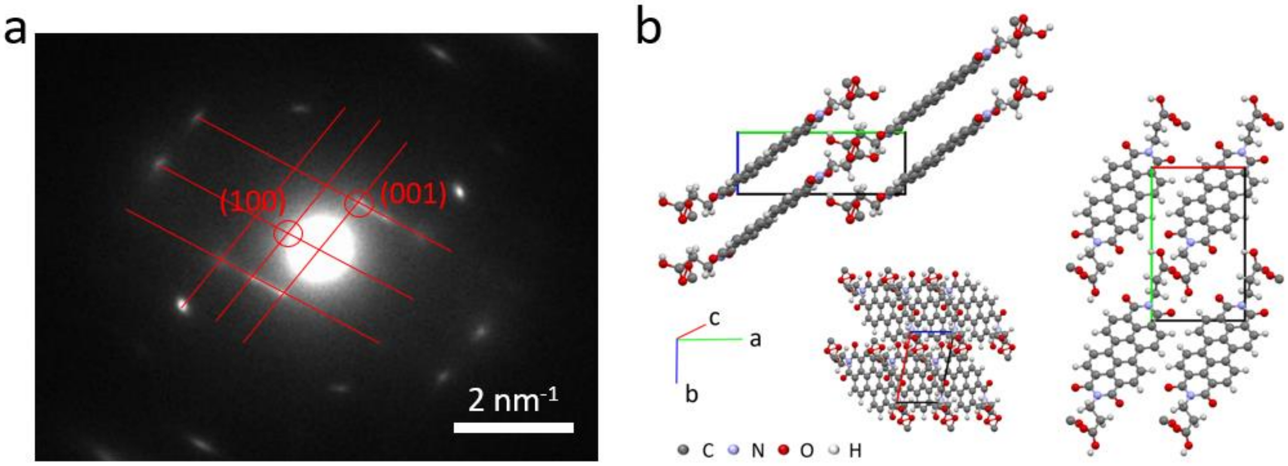

Supplementary Fig. 14. The crystal structure of PDI. (a) Single-crystal SAED pattern of PDI. **(b)** A simulated arrangement model of PDI crystal according to the SAED and XRD patterns.

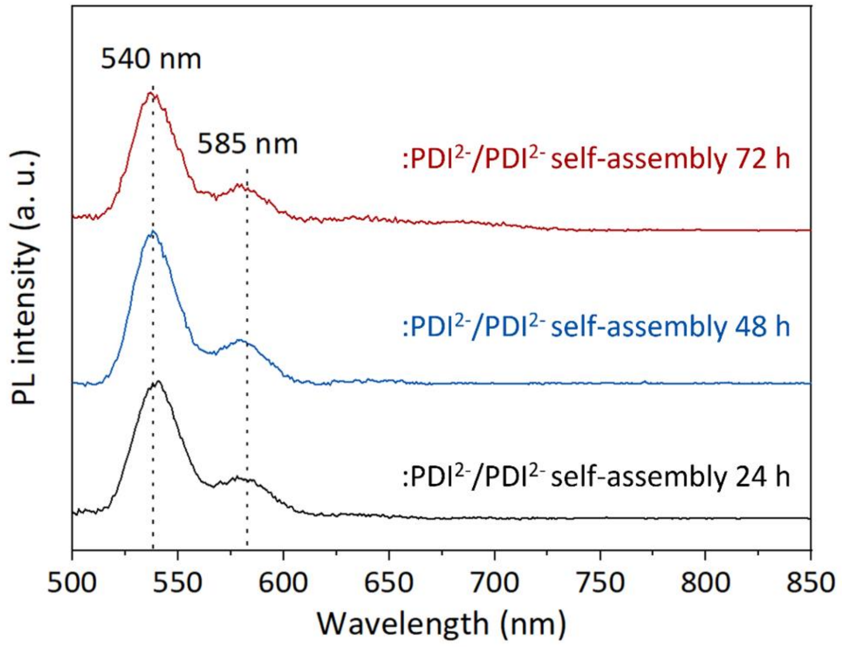

Supplementary Fig. 15. Comparison of photoluminescence under different reaction conditions. PL spectra (λ_{ex}=450 nm) of :PDI^{2-}/PDI^{2-} (solid) with different self-assembly times.

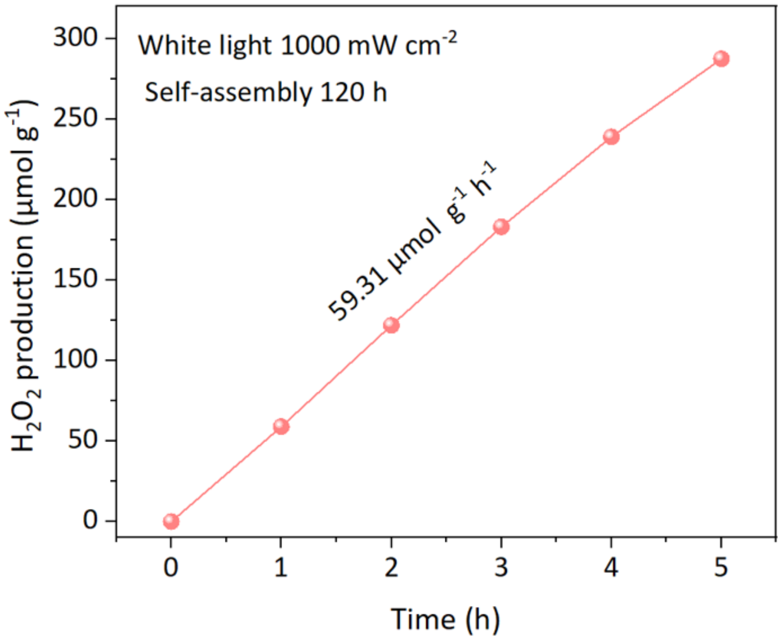

Supplementary Fig. 16. H_2O_2 production test during the photocatalytic overall water splitting. Time-profile of H_2O_2 production during the overall water-splitting experiment on :PDI^{2-}/PDI^{2-} (120 h) catalyst.

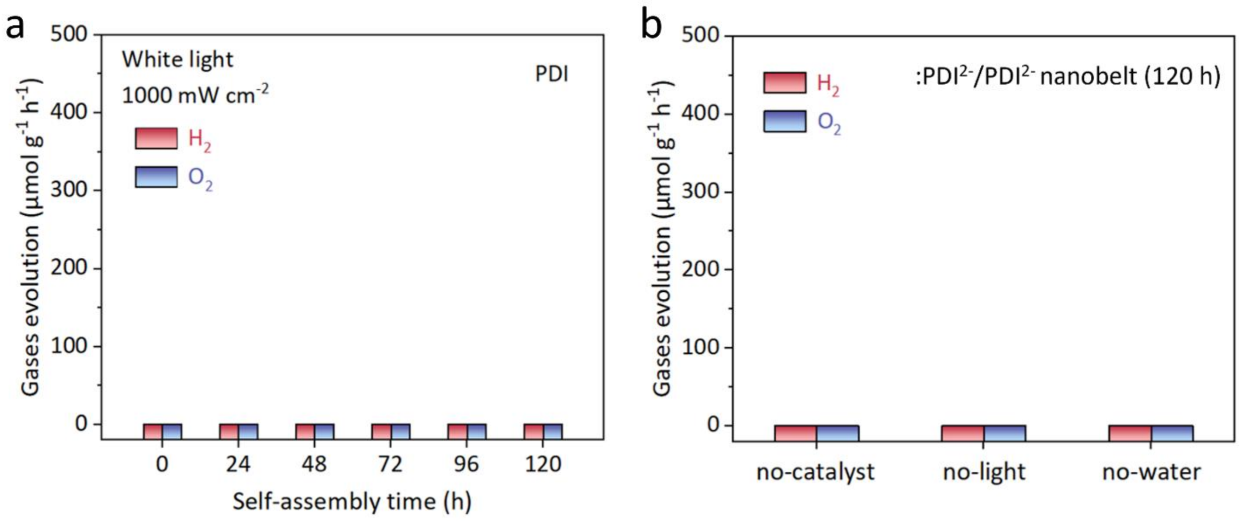

Supplementary Fig. 17. Comparative experiment on photocatalytic overall water splitting. (a) Water-splitting performances on PDI with different self-assembly time. **(b)** Water-splitting experiments without catalysts, light or H_2O over :PDI^{2-}/PDI^{2-} nanobelt (120 h) catalyst.

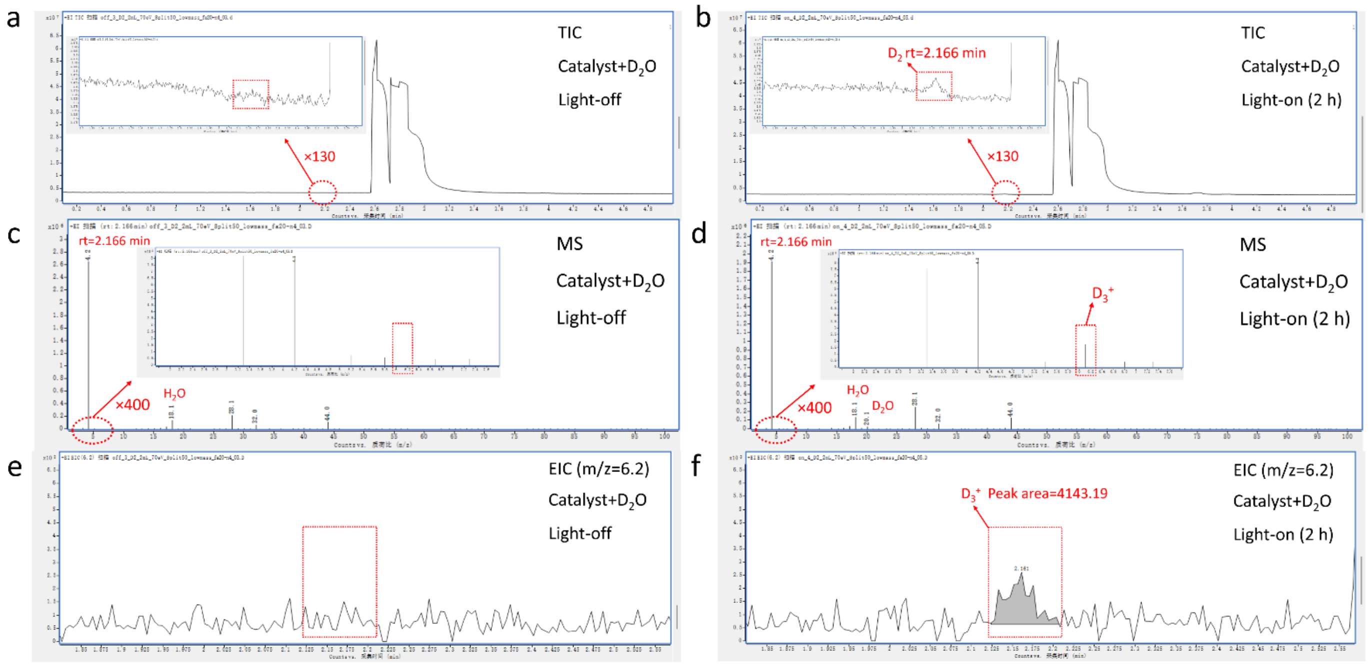

Supplementary Fig. 18. GC-MS analytical data using Agilent CP-Molsieve 5A column at 70 eV for D isotope-tracing experiments in D2O overall splitting using catalysts (catalyst/D2O=20 mg/10 mL). The TIC of 2 mL products generated by **(a)** light-off and **(b)** light-on for 2 h (inset shows the 130-fold amplification of y-axis scale), the corresponding MS spectra (RT=2.166 min) of 2 mL products generated by **(c)** light-off and **(d)** light-on for 2 h (inset shows the 400-fold amplification of y-axis scale), EIC (m/z=6.2) of 2 mL products generated by **(e)** light-off and **(f)** light-on for 2 h.

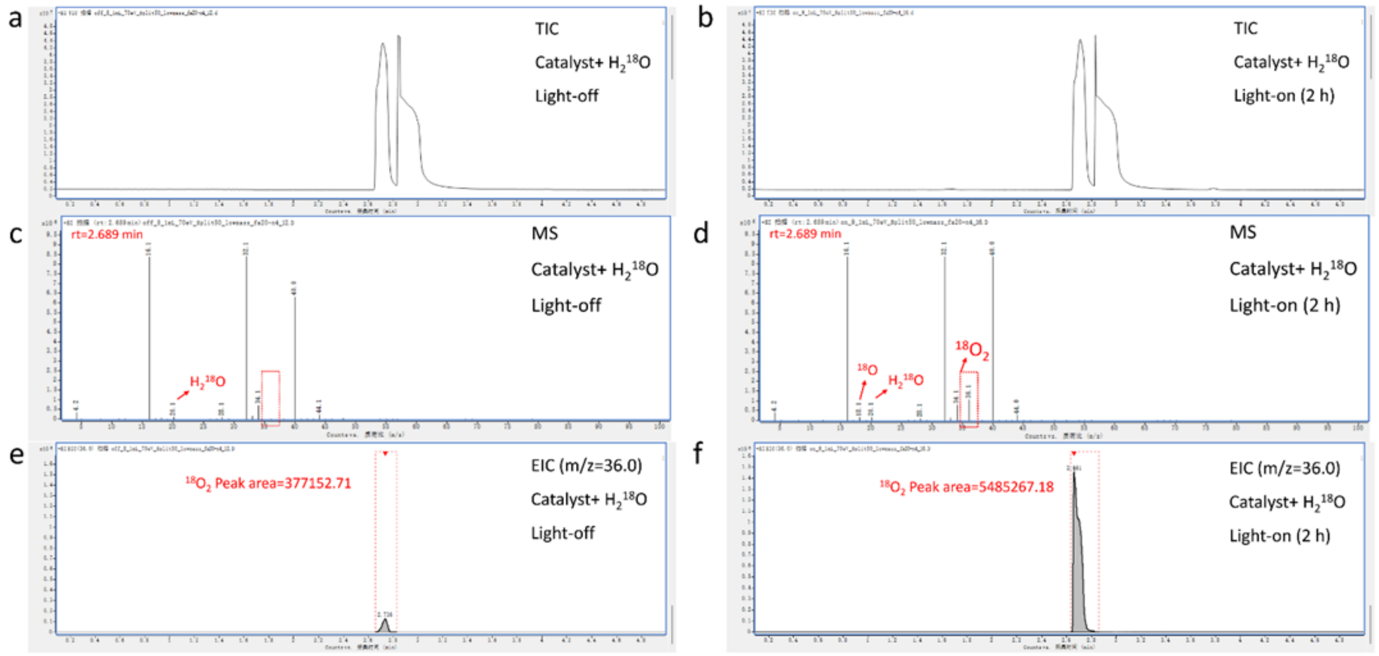

Supplementary Fig. 19. GC-MS analytical data using Agilent CP-Molsieve 5A column at 70 eV for ^{18}O isotope-tracing experiments in $H_2{}^{18}O$ overall splitting using catalysts (catalyst/$H_2{}^{18}O$ =20 mg/10 mL). The TIC of 1 mL products generated by **(a)** light-off and **(b)** light-on for 2 h, the corresponding MS spectra (RT=2.689 min) of 1 mL products generated by **(c)** light-off and **(d)** light-on for 2 h, EIC (m/z=36.0) of 1 mL products generated by **(e)** light-off and **(f)** light-on for 2 h.

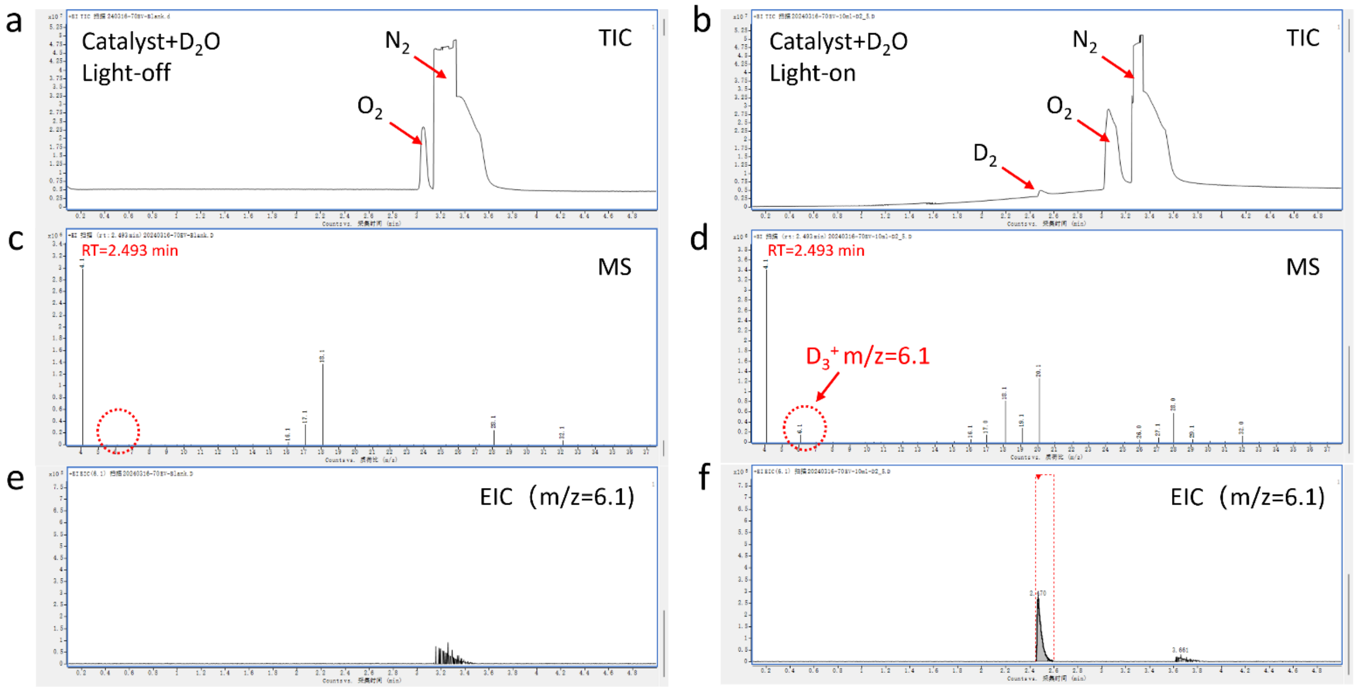

Supplementary Fig. 20. GC-MS analytical data using headspace vial under 70 eV for D isotope-tracing experiments in D_2O overall splitting using catalysts (catalyst/D_2O=20 mg/2 mL). The TIC of products generated by (a) light-off and (b) light-on for 10 h, the corresponding MS spectra (RT=2.493 min) of products generated by (c) light-off and (d) light-on for 10 h, EIC (m/z=6.1) of products generated by (e) light-off and (f) light-on for 10 h.

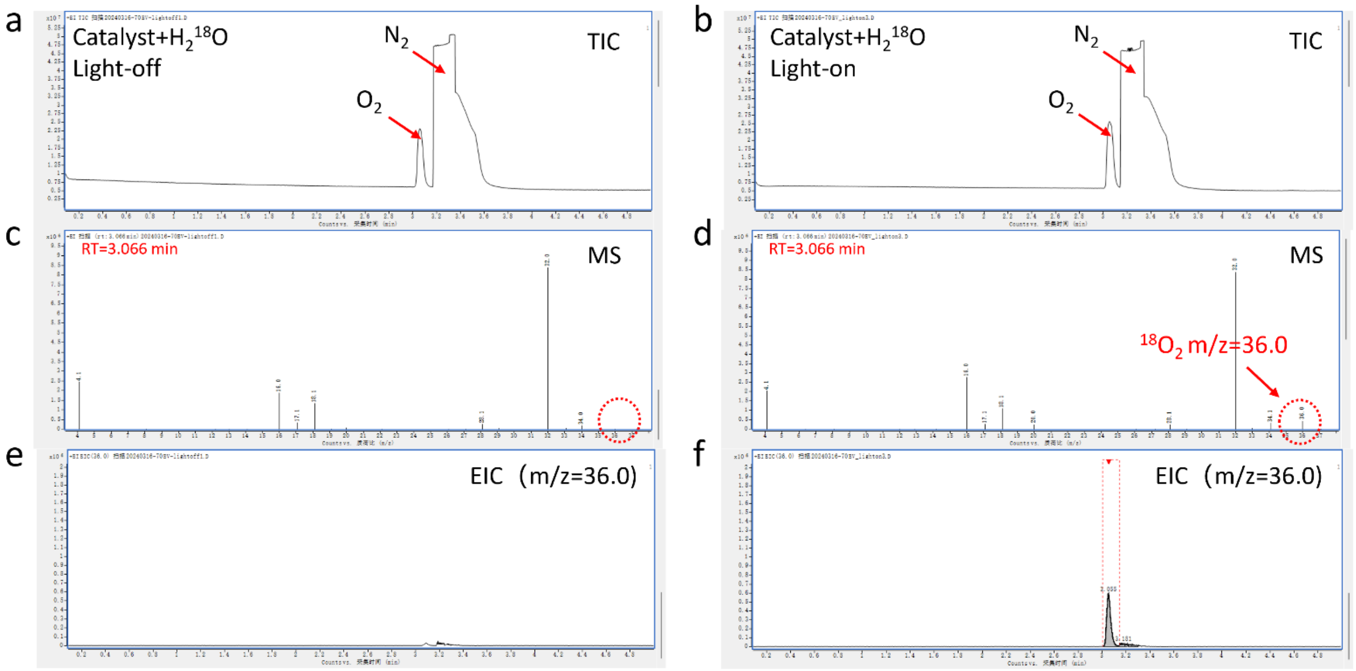

Supplementary Fig. 21. GC-MS analytical data using headspace vial under 70 eV for ^{18}O isotope-tracing experiments in $H_2{}^{18}O$ overall splitting using catalysts (catalyst/$H_2{}^{18}O$ =20 mg/2 mL). The TIC of products generated by (a) light-off and (b) light-on for 2 h, the corresponding MS spectra (RT=3.066 min) of products generated by (c) light-off and (d) light-on for 2 h, EIC (m/z=36.0) of products generated by (e) light-off and (f) light-on for 2 h.

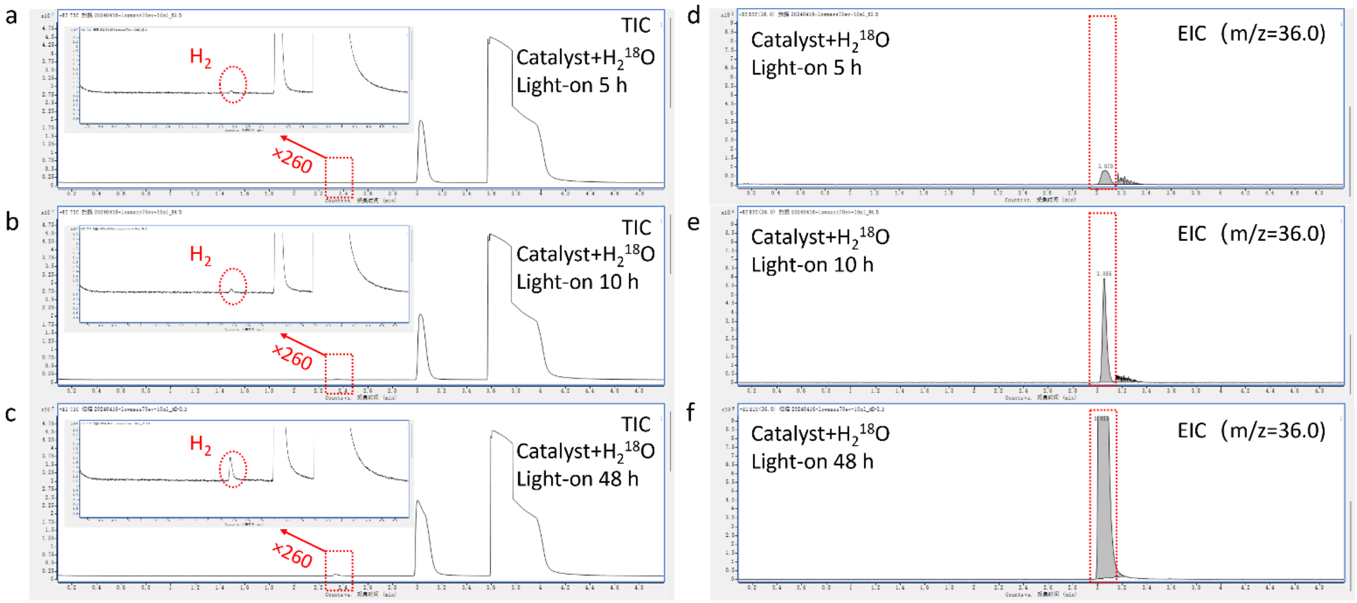

Supplementary Fig. 22. GC-MS analytical data using headspace vial under 70 eV for ^{18}O isotope-tracing experiments in $H_2^{18}O$ overall splitting using catalysts (catalyst/$H_2^{18}O$ =20 mg/2 mL). The TIC of products generated by (a) light-on for 5 h, (b) light-on for 10 h and (c) light-on for 48 h (inset shows the 260-fold amplification of y-axis scale), EIC (m/z=36.0) of products generated by (d) light-on for 5 h, (e) light-on for 10 h and (f) light-on for 48 h.

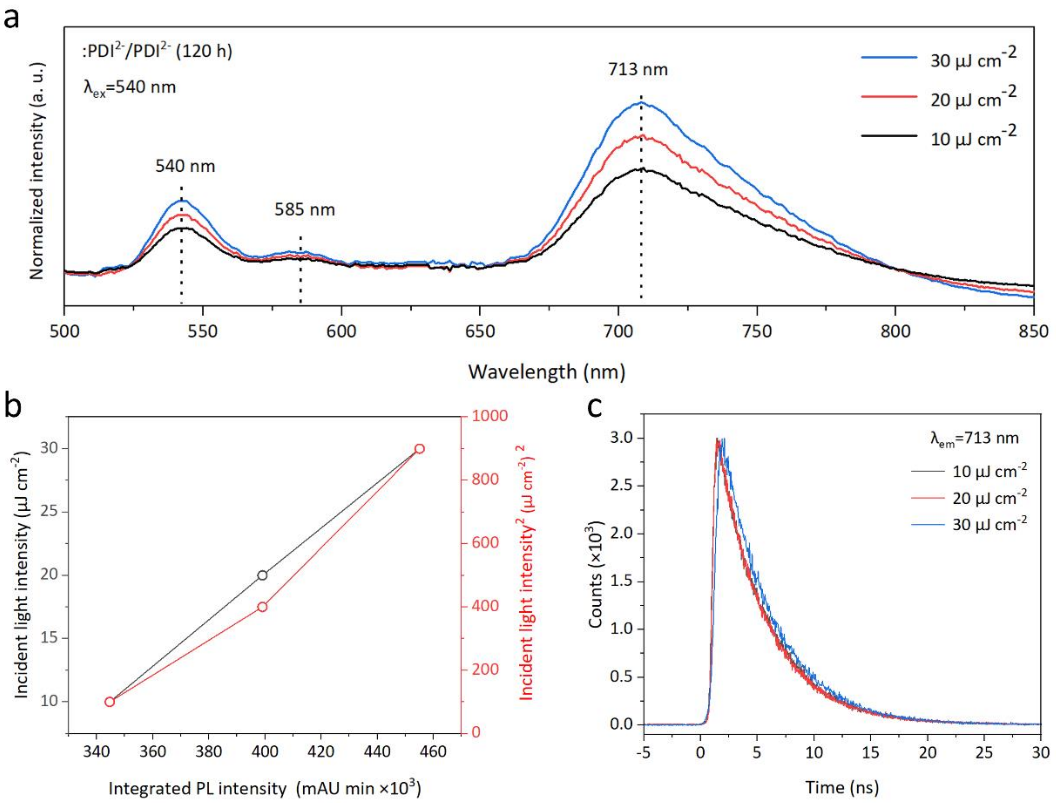

Supplementary Fig. 23. Control experiments show PL spectra under different conditions. (a) Intensity dependent PL spectra (λ_{ex}=450 nm) on :PDI^{2-}/PDI^{2-} (120 h) sample (10 μJ cm^{-2}~30 μJ cm^{-2}). **(b)** The linear relationship between integrated PL intensity and incident light intensity (and the square I^2). **(c)** Intensity dependent transient PL decay profile of λ_{em}=713 nm emission (λ_{ex}=450 nm) on :PDI^{2-}/PDI^{2-} (120 h).

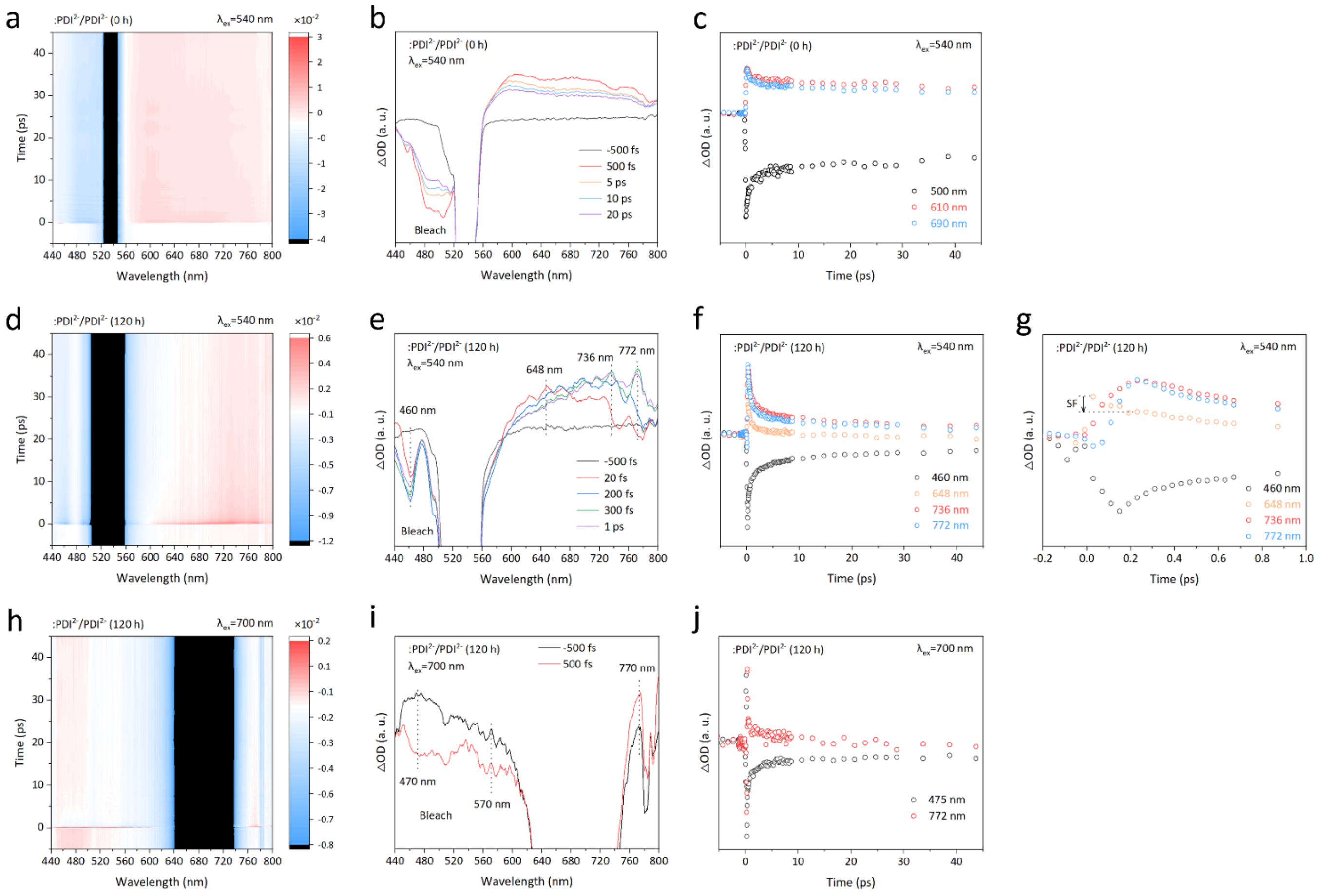

Supplementary Fig. 24. Femtosecond transient absorption. 2D map (left), spectral traces for selected time point (middle) and TA kinetic traces extracted under different wavelength (right) **(a)-(c)** :PDI^{2-}/PDI^{2-} (0 h), λ_{ex}=540 nm, **(d)-(g)** :PDI^{2-}/PDI^{2-} (120 h), λ_{ex}=540 nm and **(h)-(j)** :PDI^{2-}/PDI^{2-} (120 h), λ_{ex}=700 nm.

Fs-TAS data analysis and signal assignment: As illustrated in Supplementary Fig. 24a and b, for the :PDI^{2-}/PDI^{2-} precursor sample before self-assembly, excited state absorption (ESA) bands at 610 nm and 690 nm are observed, along with a negative bleach band from 450 to 500 nm upon λ_{ex}=540 nm excitation. The kinetic profiles of the bleach and ESA bands exhibit a mirror-like trend (Supplementary Fig. 24c), suggesting that no other transitions between ESA features occur. However, for the 120 h self-assembled :PDI^{2-}/PDI^{2-} nanobelt sample, the bleach signal shifts to 470 nm due to different ground states, and additional ESA features appear at 736 nm and 772 nm (Supplementary Fig. 24d and e). These features display slower generation dynamics and longer lifetimes compared to the original ESA feature at 648 nm (Supplementary Fig. 24f). We attribute the ESA feature at 648 nm to the singlet excited state after direct excitation, while the newly emerged ESA features at 736 nm and 772 nm are likely triplet states resulting from singlet fission (SF). The opposing growth trends of the singlet and triplet ESA features from 20 fs to 300 fs (Supplementary Fig. 24g) further support

our assignment and the occurrence of the SF process, with the SF yield calculated to be approximately 42%. Upon λ_{ex}=700 nm excitation, bleach signals are observed at 470 nm and 570 nm, and the singlet ESA from 600 to 650 nm is no longer detectable. Instead, an emerging ESA feature at 770 nm is clearly observed (Supplementary Fig. 24h-j), providing direct evidence for the S_0-T_1 transition.

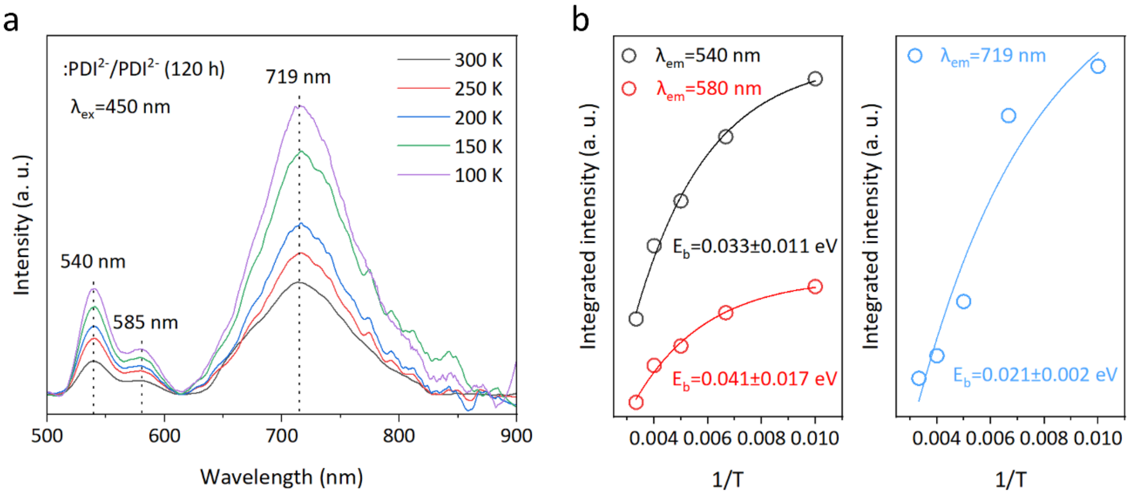

Supplementary Fig. 25. The binding energy (*E_b*) of *S_1 singlet excitons after the triplet fusion upconversion (TFU) process. (a) Temperature-dependent PL emission spectra (λ_{ex}=450 nm) of :PDI^{2-}/PDI^{2-} catalyst (120 h) in the temperature range of 100 K~300 K. **(b)** Integrated PL intensity from (a) as a function of temperature. E_b is calculated by Arrhenius plot fittings.

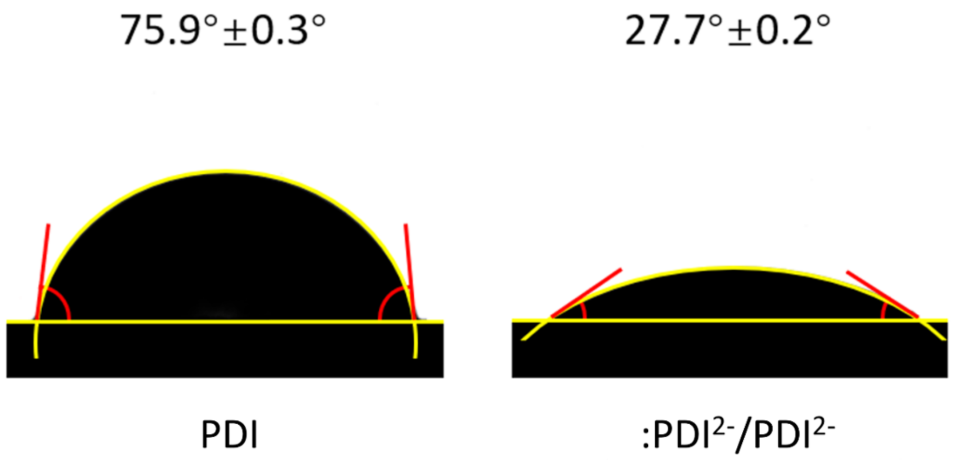

Supplementary Fig. 26. Comparison of hydrophilicity. The water contact angle measurement on PDI and :PDI^{2-}/PDI^{2-}.

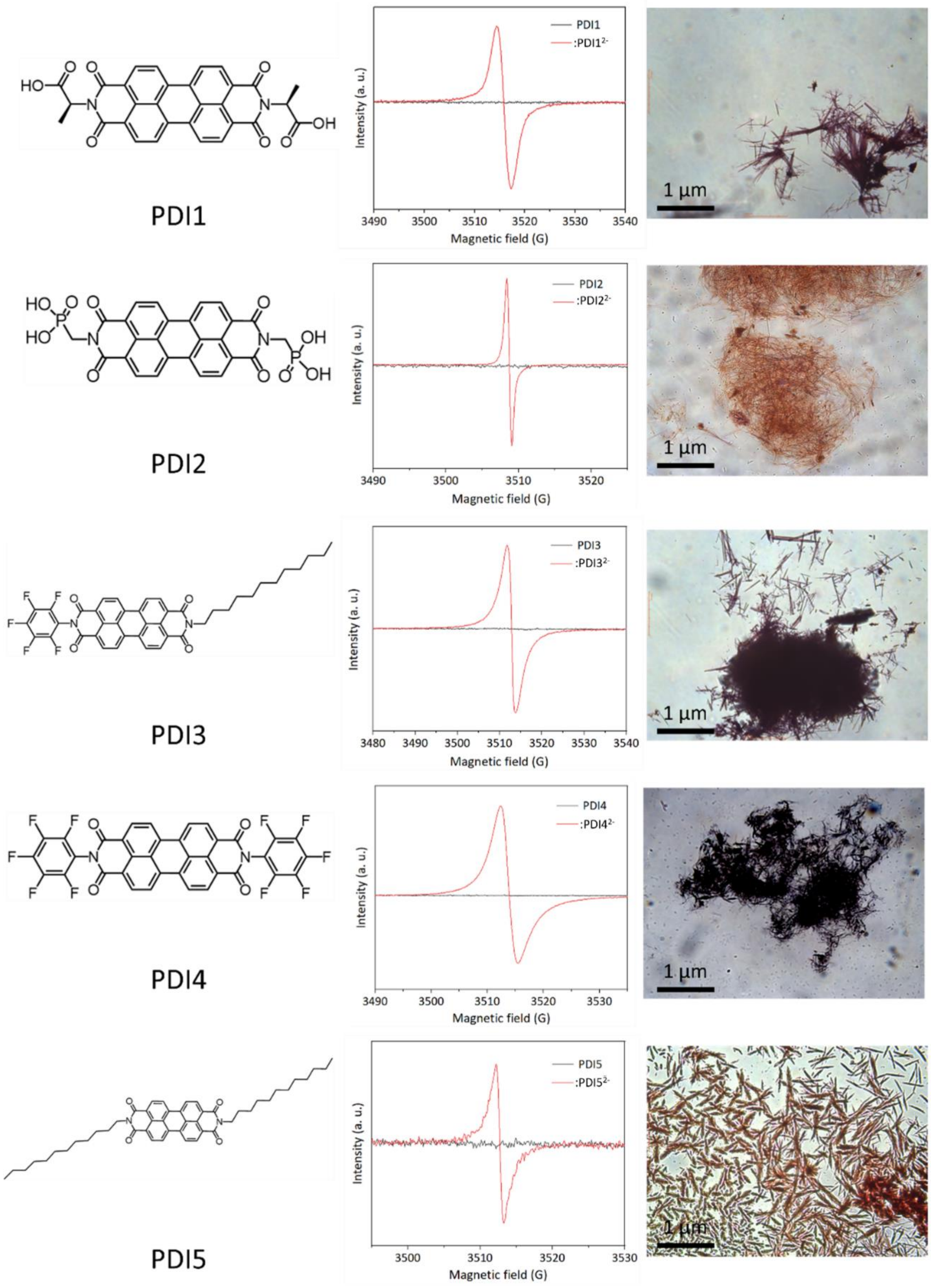

Supplementary Fig. 27. Attempts for follow-up work on the PDI family. Radical self-assembly strategy applies to five PDI derivatives (PDI1-PDI5) with ESR spectra and optical microscope photos.

Supplementary Table 1. Crystal parameters of PDI and :PDI^{2-}/PDI^{2-}.

Sample	length of a-axis (Å)	length of b-axis (Å)	length of c-axis (Å)
PDI	14.00	5.25	8.69
:PDI^{2-}/PDI^{2-}	16.80	8.29	6.40

Supplementary Table 2. Wavelength-dependent AQYs and corresponding H_2 production amount on :PDI^{2-}/PDI^{2-} nanobelt (120 h).

Wavelength (nm)	H_2 evolution (μmol)	Light intensity (mW cm^{-2})	Irradiation area (cm^2)	Irradiation time (h)	AQY (%)
365	0	23.7	4	5	0
420	12.5	236.6	4	5	0.4247
425	26.2	241.5	4	5	0.8494
450	37.6	275.2	4	5	1.0138
475	45.9	266	4	5	1.1097
500	43.8	240.7	4	5	1.2193
530	56.8	195.2	4	5	1.5755
550	87.5	269.8	4	5	1.9591
580	61.3	210.5	4	5	1.6714
600	38.3	166.9	4	5	1.2741
630	56.1	201.2	4	5	1.4796
650	45.3	154.7	4	5	1.5344
670	46.4	143.1	4	5	1.6166
700	28.4	100.5	4	5	1.3426
725	26.9	99.5	4	5	1.2467
760	28.2	102.7	4	5	1.2056
850	18.7	80.2	4	5	0.9453

Supplementary Table 3. Wavelength-dependent AQYs and corresponding H_2 production amount on :PDI^{2-}/PDI^{2-} nanorod (24 h).

**Wavelength (nm)**	**H_2 evolution (μmol)**	**Light intensity (mW cm^{-2})**	**Irradiation area (cm^2)**	**Irradiation time (h)**	**AQY(%)**
365	0	15.9	4	5	0
420	3.3	154.2	4	5	0.17
425	5.3	220.5	4	5	0.19
450	7.8	253.2	4	5	0.23
475	9.7	260	4	5	0.26
500	12.9	244.7	4	5	0.35
530	12.4	200	4	5	0.39
550	17.4	250.7	4	5	0.42
580	10.4	170.5	4	5	0.35
600	7.6	155	4	5	0.27
630	5.2	180.7	4	5	0.16
650	2.7	100.8	4	5	0.13
670	2.6	131.1	4	5	0.1
700	1.7	98	4	5	0.08
725	1.3	82.7	4	5	0.072
760	0.8	69.5	4	5	0.05
850	0.6	70.2	4	5	0.034

Supplementary Table 4. AQYs and corresponding H_2 production amount on :PDI^{2-}/PDI^{2-} with different self-assembly time under λ=550 nm illumination.

**Self-assembly time (h)**	**H_2 evolution (μmol)**	**Light intensity (mW cm^{-2})**	**Irradiation area (cm^2)**	**Irradiation time (h)**	**AQY(%)**
0	0.99	200	4	5	0.03
24	23.8	200	4	5	0.72
48	31.4	200	4	5	0.95
72	43.6	200	4	5	1.32
96	59.2	200	4	5	1.79
120	64.8	200	4	5	1.96
